# Cell line name recognition in support of the identification of synthetic lethality in cancer from text

**DOI:** 10.1093/bioinformatics/btv570

**Published:** 2015-10-01

**Authors:** Suwisa Kaewphan, Sofie Van Landeghem, Tomoko Ohta, Yves Van de Peer, Filip Ginter, Sampo Pyysalo

**Affiliations:** 1 ^1^Turku Centre for Computer Science (TUCS), 20520 Turku, Finland,; 2 ^2^Department of Information Technology, University of Turku, 20014, Finland,; 3 ^3^University of Turku Graduate School (UTUGS), University of Turku, 20014, Finland,; 4 ^4^Department of Plant Systems Biology, VIB, Ghent 9000, Belgium,; 5 ^5^Department of Plant Biotechnology and Bioinformatics, Ghent University, Ghent 9052, Belgium,; 6 ^6^Textimi, Tokyo, Japan,; 7 ^7^Bioinformatics Institute Ghent, Ghent University, Ghent, Belgium,; 8 ^8^Genomics Research Institute, University of Pretoria, Pretoria, South Africa and; 9 ^9^Language Technology Lab (LTL), University of Cambridge, Cambridge CB3 9DA, United Kingdom

## Abstract

**Motivation:** The recognition and normalization of cell line names in text is an important task in biomedical text mining research, facilitating for instance the identification of synthetically lethal genes from the literature. While several tools have previously been developed to address cell line recognition, it is unclear whether available systems can perform sufficiently well in realistic and broad-coverage applications such as extracting synthetically lethal genes from the cancer literature. In this study, we revisit the cell line name recognition task, evaluating both available systems and newly introduced methods on various resources to obtain a reliable tagger not tied to any specific subdomain. In support of this task, we introduce two text collections manually annotated for cell line names: the broad-coverage corpus Gellus and CLL, a focused target domain corpus.

**Results:** We find that the best performance is achieved using NERsuite, a machine learning system based on Conditional Random Fields, trained on the Gellus corpus and supported with a dictionary of cell line names. The system achieves an F-score of 88.46% on the test set of Gellus and 85.98% on the independently annotated CLL corpus. It was further applied at large scale to 24 302 102 unannotated articles, resulting in the identification of 5 181 342 cell line mentions, normalized to 11 755 unique cell line database identifiers.

**Availability and implementation:** The manually annotated datasets, the cell line dictionary, derived corpora, NERsuite models and the results of the large-scale run on unannotated texts are available under open licenses at http://turkunlp.github.io/Cell-line-recognition/.

**Contact:** sukaew@utu.fi

## 1 Introduction

Biomedical text mining methods are increasingly capable of accounting for the diversity of information found in this domain. While proteins and their interactions received much attention in BioNLP research in the last decade (Krallinger *et* *al.*, 2007; Pyysalo *et* *al.*, 2008; Tikk *et* *al.*, 2010; Tsuruoka and Tsujii, 2003), recent efforts have increasingly focused on complex structured extraction with targets such as general regulatory associations and gene expression (Kim *et* *al.*, 2011), post-translational modifications and epigenetics (Pyysalo *et* *al.*, 2012), pathway construction (Ohta *et* *al.*, 2013) and a variety of other biological processes (Björne and Salakoski, 2013; Miwa and Ananiadou, 2013; Pyysalo *et* *al.*, 2013).

However, one important category of associations that has received comparatively little attention so far consists of *functional* interactions between gene products. Two genes in a functional interaction can, for instance, be associated to a specific disease or condition, or a particular phenotype. An example of such a case is a pair of synthetically lethal (SL) genes, for which a mutation in one of the two does not cause loss of viability, but the simultaneous inhibition of both genes leads to cell death (Brough *et* *al.*, 2011). Through the identification of SL interaction pairs, it would for instance be possible to target specific tumours that have limited pharmacological tractability.

One approach to identify SL interactions from the literature is by analyzing studies in which a certain gene is found to be lethal in a specific cell line. All known somatic mutations in that cell line can then be combined with the gene found in the literature to form candidate SL pairs. Often, the known somatic mutations of a specific cell line are not mentioned in the article, so a crucial step involves the normalization of a specific cell line symbol from text to its standardized database identifier in authoritative resources such as Cellosaurus (http://web.expasy.org/cellosaurus/), CCLE (Barretina *et* *al.*, 2012), COSMIC (Forbes *et* *al.*, 2011) or CLDB (Romano *et* *al.*, 2009).

As cell lines play an important role in biomedical research, they have attracted great interest from the text-mining community. Several corpora such as GENIA (Kim *et* *al.*, 2003), AnEM (Ohta *et* *al.*, 2012) and CellFinder (Neves *et* *al.*, 2012) have included cell line mentions among their annotation targets. Further, numerous automated tools have been developed to recognize cell lines from text. Notably, systems participating in the 2004 JNLPBA Shared Task were required to recognize cell line mentions among other targets in their named entity recognition (NER) challenge (Kim *et* *al.*, 2004). The best performance at this part of the task was achieved by a machine learning (ML) approach with 59.23% ***F*-**score (Zhou and Su, 2004). In addition to ML approaches, dictionary-based methods have also been used for cell line name recognition, achieving an ***F*-**score of 69% on the recently introduced CellFinder corpus (Neves *et* *al.*, 2013).

There is significant variance in results reported for cell line name recognition tools, making it challenging to choose a suitable NER system for real-world tasks where reliable, broad-coverage recognition and normalization of cell line names in text is required. In addition, the lack of the ability to link mentions to external resources has limited the usability of the cell line taggers. In this study, we consider a variety of available resources and tools to identify the most promising approach to recognize cell line names from text, and assess differences between task definitions, methods and annotated resources. To support this effort, we annotated two corpora and release them for public use: Gellus, a broad domain cell line annotation corpus used for training and testing, and CLL, an independent evaluation corpus for established cell line mentions.

We additionally implemented a method for normalizing the tagged cell line names to a controlled cell line vocabulary, Cellosaurus. Following the identification of the best recognition and normalization approaches, we applied these to all PubMed abstracts and PubMed Central full-text documents to identify and normalize cell line name mentions in the entire publicly available literature.

## 2 Approach

In this section, we describe our methodology in more detail. First, we provide the specific definition of our cell line mention recognition task (Section 2.1). We then describe publicly available cell line corpora, as well as those that are newly annotated in this work (Section 2.2). Further, we outline the available NER tools that recognize cell line names (Section 2.3). Finally, we implement a normalization procedure to map ambiguous textual symbols of cell lines to their unique identifiers in external resources (Section 2.4).

### 2.1 Task definition

We firstly scope our task of cell line mention recognition by defining what is considered a cell line. Following the definition of Cell Line Ontology (CLO) (Sarntivijai *et* *al.*, 2011), we define *cell line* as *a genetically stable and homogenous population of cultured cells that shares a common propagation history* via experimental and selection processes. Cell lines can thus establish uniform and stable populations that maintain their characteristics over long periods of time, even indefinitely. Consequently, non-specific mentions such as ‘HUVECs’ are not considered cell lines in our work, while mentions such as ‘HUVEC-C’ are.

Secondly, we consider it a crucial property of a cell line NER system to recognize specifically those mentions in text that can be unambiguously linked to *established cell line names.* This is important for instance for the application to the identification of SL pairs, as the identified cell lines from text need to be characterized with their known mutations by consulting external resources. As a result, mentions such as ‘cancer cell line’ or ‘endothelial cell line’, which might be useful in other applications, are considered too generic and thus excluded from this study.

### 2.2 Data

Here, we briefly describe a cell line dictionary we constructed by integrating information from various authoritative cell line resources. Further, we describe two publicly available annotated corpora, JNLPBA and CellFinder, and we finally present those newly created in this study, Gellus and CLL.

#### 2.2.1 Cell line dictionary

We gathered a dictionary of cell line names derived from the Cellosaurus resource (version 6.5). We extracted all cell line names (e.g. *GOS-3*), accessions (e.g. *CVCL_2050*), synonyms (e.g. *GOS3*), as well as non-ambiguous identifiers to external resources such as CCLE or CLDB (e.g. *cl5278*) from Cellosaurus. Additionally, we augmented this data with mutation information obtained from CCLE and Cosmic, which are specific for human cancer cell lines, our application domain of interest for the envisioned use-case on SL gene pairs.

In total, the dictionary contains 89 446 strings linked to 26 731 unique cell lines from 151 organisms. Within this set,  1174 cancer cell lines can be associated to known somatic mutations.

#### 2.2.2 JNLPBA

The JNLPBA corpus was created for the JNLPBA shared task (Kim *et* *al.*, 2004) based on the GENIA corpus (Kim *et* *al.*, 2003). The corpus consists of 2404 abstracts, divided into a training set of 2000 documents and a test set of 404 documents. The corpus is annotated for mentions of physical entities of five types: cell line, cell type, dna, rna and protein. Like its source corpus, GENIA, the JNLPBA corpus consists of documents drawn from the relatively restricted subdomain of *transcription factors in human blood cells.*

In the present study, we consider only the cell line mention recognition subtask of JNLPBA, filtering the corpus to remove annotations of types other than cell line. We further randomly divide the original training set into training and development subsets for parameter selection, selecting 1500 documents for the new training set and 500 for the new development set. The resulting filtered corpus, JNLPBA_cl__,_ contains 4330 cell line annotations.

#### 2.2.3 CellFinder

The CellFinder corpus (version 1.0) (Neves *et* *al.*, 2012) contains annotations of 10 specifically selected full-text articles (2177 sentences) from the *human embryonic stem cells* domain. The annotations mark six types of entities: anatomical part, cell component, cell line, cell type, gene/protein and species. (The corpus was recently extended to kidney stem cell articles, introducing annotation for gene expression (Neves *et al.*, 2013). However, as this version was not publicly available during our study, we used CellFinder version 1.0 in our work.)

The corpus contains 5275 entity annotations, of which 440 are cell line. The annotations were created by two domain experts, and the released corpus was created by merging both consensus and distinct annotations from the two annotators, leading to some overlapping annotations in the data.

We prepared a filtered version of the corpus, CellFinder_cl__,_ by keeping only cell line annotations and discarding overlapped annotations, resulting in 386 annotations. We divided the corpus into a training set of seven documents and a test set of three documents. (Specifically, we modified the 50/50% corpus split introduced by Neves *et al.* (2012), adding two documents (PMIDs 15971941 and 16672070) to the training set to balance the annotation distribution and increase the size of the training data for machine learning.)

#### 2.2.4 Gellus

We created the *Ge*nt ce*ll*-line corp*us* (Gellus) by annotating cell line names in 1212 documents drawn from PubMed abstracts and PMC full text extracts. The documents were annotated to identify the names of specific cell lines or established categories of cell lines (Section 2.1). Only the spans of the actual names were marked, not including premodifiers or head nouns such as ‘cells’. Half of the corpus texts were drawn from the AnEM corpus (Ohta *et* *al.*, 2012), a collection of randomly selected PubMed abstracts and full paper extracts previously annotated for mentions of anatomical entities. The other half was drawn from the BioNLP ST’13 Cancer Genetics (CG) task documents (Pyysalo *et* *al.*, 2013), a subset of PubMed abstracts in the cancer genetics domain previously annotated to identify mentions of anatomical and molecular entities and events. The documents thus cover both a random subset of the literature and a focused sample of cancer documents.

The Gellus annotation was performed by a biologist with extensive experience in biomedical domain annotation. The brat annotation tool (Stenetorp *et* *al.*, 2012) was used for the human annotation work. The Gellus annotation effort identified 650 cell line mentions. An inter-annotator agreement analysis was carried out by another biologist annotating 100 randomly selected documents consisting of 84 tokens tagged as being part of a cell line name and 5212 ‘negative’ tokens not tagged as part of a name. Token-level inter-annotator agreement for this portion of documents is 99.8% accuracy, with Cohen’s kappa score of 0.9432. The very high accuracy largely reflects agreement on the extremely common negative class label. Alternatively, the inter-annotator ***F*-**score is 93.85%, still a high level of agreement. We divide the corpus documents into 50/17/33% training/development/test sets, stratified to maintain equal distributions of random and cancer domain documents in the subsets.

#### 2.2.5 CLL corpus

To allow for an extrinsic evaluation of the recognition of unambiguous cell line names from text, we annotated a balanced sample of sentences containing names from the cell line dictionary (Section 2.2.1). To avoid bias toward a limited number of well-known and well-described cell lines, we first sampled 3000 cell names at random from the dictionary. For each sampled name, we then selected at random a PubMed citation or a PubMed Central Open Access (PMC-OA) full-text article that contained that specific name, using strict, case-sensitive matching criteria and ensuring that no single document was chosen twice for distinct names. Approximately 15% of the names were matched in the literature, resulting in an initial dataset of 454 documents with exactly one tagged candidate cell line name each.

We then manually evaluated a randomly selected subset of 201 documents, marking whether the candidate name did in fact represent a cell line. For candidates that were not cell lines, the correct entity type (e.g. gene/protein or organism) was marked. We simultaneously annotated all other cell lines names occurring in the same sentence as the candidate mention. This *C*el*l l*ine corpus (CLL) was used for the open-domain evaluation described in Section 3.4.

### 2.3 NER tools

To recognize cell line names in text, we consider both dictionary-based tagging and selected publicly available NER tools that are applicable to the task. These tools include ABNER (Settles, 2005), GENIA tagger (Tsuruoka *et* *al.*, 2005) and Gimli (Campos *et* *al.*, 2013), trained on the JNLPBA corpus. Additionally, we apply the retrainable NERsuite (http://nersuite.nlplab.org/) system on our newly annotated training corpus Gellus (Section 2.2.4). We compare the performance of these methods on various corpora using their held-out test sets.

To accommodate for minor differences in the extent of annotated spans in the different corpora, the evaluation criteria applied in this study and reported throughout the manuscript accept any overlap between a cell line mention tagged by a system and a gold standard annotation as a match.

#### 2.3.1 Dictionary look-up

We perform pattern matching using the cell line dictionary (Section 2.2.1) against all three corpora as a baseline for comparison with ML-based methods. Dictionary-based tagging is performed using two matching-specificity criteria: ***exact matching***, aiming for high precision, and ***approximate matching***, for high recall. The ***approximate criterion*** considers strings to match regardless of case and additionally only requires alpha-numerical characters to match; all other characters, such as space and hyphen are ignored. Thus, for example *gos3* matches both *GOS 3* and *Gos-3* under the approximate matching criterion.

#### 2.3.2 ABNER

The supervised ML-based tagger ABNER (Settles, 2005), is implemented using Conditional Random Fields (CRFs) (Lafferty *et* *al.*, 2001) with orthographic and contextual feature sets (Settles, 2004). The system is distributed with models trained on two corpora, BioCreative and JNLPBA, allowing the tagging of various types of bio-entities, including cell lines.

ABNER provides a graphical user interface with a variety of features including automatic tokenization, batch mode annotation and a Java API which allows training ABNER on new corpora (Settles, 2005). We use the system out-of-the-box with the built-in JNLPBA model to detect cell lines.

#### 2.3.3 GENIA Tagger

The integrated GENIA tagger system provides various levels of text analysis: part-of-speech (POS) tagging, text chunking and NER (Tsuruoka *et* *al.*, 2005). The tagging is based on a maximum entropy classifier and a bidirectional inference algorithm (Tsuruoka and Tsujii, 2005).

For POS tagging, the system is specifically tuned for analyzing English biomedical text, as it is trained on a combination of corpora from both biomedical and newspapers domains. For NER, the tagger is trained on the JNLPBA corpus, and it can thus recognize cell lines along with the other four JNLPBA entity types.

We use auto-tokenization, and apply the tagger with default settings. Note that the GENIA tagger does not provide tools for training on new corpora.

#### 2.3.4 Gimli

To recognize various types of biomedical entities including cell lines, Gimli implements linguistic analysis with supervised ML (Campos *et* *al.*, 2013). The tagging component is based on CRFs and trained on the GENETAG and JNLPBA corpora, complemented with external lexicons and biomedical term resources. The best model provided with Gimli for cell line mention detection is a second-order CRF model trained on the JNLPBA corpus.

Gimli also provides the possibility of training the system with new corpora. However, as the system is distributed with a model tuned by the authors for cell line name detection and we are interested in the performance of the system in general, we only used the provided model with default settings in this study.

#### 2.3.5 NERsuite

NERsuite is a generic named entity recognition toolkit based on the CRFsuite (http://www.chokkan.org/software/crfsuite/) (Okazaki, 2007) implementation of CRFs. It defines a broad set of features that are known to be beneficial for entity mention recognition tasks, including features based on the token surface form, lemma, POS tagging, shallow parsing and orthography. The toolkit has previously been shown to achieve competitive performance in biomedical domain entity mention detection tasks (Campos *et* *al.*, 2013).

For NERsuite, we trained new models on all corpora, selecting the regularization and label bias parameters using a grid search of parameter values and evaluating performance on the development set. To assess the benefits of features derived from dictionary matching, we trained for each corpus one model with and one without the compiled cell line dictionary (Section 2.2.1), applying strict string matching against the dictionary for feature generation. For final evaluation, the system was trained on the combination of training and development sets.

### 2.4 Normalization

A crucial step following the recognition of cell line mentions in text, is their normalization or grounding, i.e. the disambiguation of occasionally ambiguous abbreviations and synonyms to unique, well-defined concepts in authoritative cell line databases such as COSMIC and CCLE.

Once the symbols are recognized from text (Section 2.3), we further link the detected mentions to Cellosaurus identifiers using both exact and approximate matching criteria (Section 2.3.1). In detail, we applied both criteria in a stepwise manner. First, we use the *exact matching approach* to map tagged cell lines to Cellosaurus names and synonyms. If none of the names or synonyms are matched, we subsequently follow an *approximate matching criterion* where the mentions along with the Cellosaurus names and synonyms are case-lowered and punctuation-stripped prior to character matching.

## 3 Results and discussion

In this section, we first provide the results of our qualitative evaluation of all available corpora (Section 3.1). For a comparative evaluation, we present the results of all tools trained on JNLPBA_cl_ in Section 3.2. We then perform evaluation with training and evaluation on additional corpora, CellFinder_cl_ and Gellus (Section 3.3), with evaluation also on the open-domain corpus CLL (Section 3.4). Finally, we apply the best-performing tool to the entire available literature and analyze the results in the framework of our application to identify SL gene pairs (Section 3.6).

### 3.1 Qualitative evaluation

We studied the corpus annotation guidelines, individual cell line annotations and annotation statistics (e.g. most frequently annotated strings) to assess qualitative differences among the corpora. We observed a number of systematic differences in the annotation, the most apparent relating to specificity constraints, the distinction between names and other mentions, and the extent of annotated spans. This section briefly presents the primary findings of this evaluation.

The specificity of annotated mentions, i.e. the degree to which a mention identifies a specific entity as opposed to a general category of entities, is closely related to the feasibility of normalizing mentions to external database resources. Mentions such as ‘MCF-7’ and ‘CHO’ that can be unambiguously linked to particular cell lines in external resources are considered specific cell line name mentions. These are the primary target of our study. By contrast, mentions such as ‘T cell line’ and ‘human monocytic cell line’ cannot be unambiguously linked to unique identifiers, and are thus insufficiently specific to qualify as cell line names in our task definition (Section 2.1). To quantify the specificity of cell line annotations in the corpora, we used approximate matching criteria (Section 2.3.1) to match each annotated string against the cell line dictionary. The results show that the JNLPBA_cl_ corpus contains the smallest portion of specific mentions (45%), compared to CellFinder_cl_ (67%) and Gellus (79%).

The newly introduced Gellus corpus only marks the minimal span of specific established individual cell lines or cell line categories, while the JNLPBA_cl_ annotation includes head nouns and various premodifiers. While most of the CellFinder cell line annotations are minimal, the corpus annotation contains a small mix of longer spans, likely resulting in part from the annotation merging. Note that for CellFinder_cl__,_ instances of annotation overlap were resolved by eliminating nested annotations (Section 2.2.3).

Table 1 summarizes the general characteristics of the corpora and selected overall statistics. In terms of the size of cell line annotations and unique strings, JNLPBA_cl_ contains the largest number of annotations as well as unique strings, followed by the Gellus and CellFinder_cl_ corpora. Both JNLPBA_cl_ and CellFinder_cl_ are specific to particular domains, while Gellus is a mixture of randomly selected articles and articles specifically selected for relevance to cancer genetics.

**Table 1.** Qualitative comparison of three corpora on different criteria

	Corpus		
Characteristics	CellFinder_cl_*	JNLPBA_cl_	Gellus
Annotation diversity**	17.10% (66/386)	58.38% (2528/4330)	32.31% (210/650)
Document size	10 full-texts	2404 abstracts	1212 documents***
Annotation span	excl. head nouns	incl. head nouns	excl. head nouns
Domain	Embryonic stem cells	blood transcription factors	random + cancer
Cell line definition	Specific	Specific + Generic	Specific
Normalized cell lines (%)	66.84 (258/386)	45.24 (1959/4330)	79.38 (516/650)

*The statistics of cell line section of the derived corpus are slightly different from the original one.

**This represents the number of unique strings per number of mentions.

***The corpus consists of 300 PubMed abstracts and extracts from 912 PMC full text documents.

### 3.2 Comparative evaluation

We evaluated the performance of the cell line taggers on the held-out test sets of the three corpora, JNLPBA_cl_ (Section 2.2.2), CellFinder_cl_ (Section 2.2.3) and Gellus (Section 2.2.4). The results are summarized in Table 2.

**Table 2.** Comparison of performance across different corpora for the overlap matching criterion

		**Test corpus (Precision/Recall/*F*-score)**		
**T**ool	**Train corpus**	**JNLPBA**_cl_	**CellFinder**_cl_	**Gellus**
Dictionary (approximate)	N/A	19.83/44.60/27.45	36.47/79.59/50.02	13.76/92.74/23.96
Dictionary (exact)	N/A	54.14/42.40/47.56	74.84/76.87/75.84	55.46/86.59/67.61
GENIA tagger	JNLPBA	66.92/69.80/68.33	20.00/23.13/21.45	40.50/55.87/46.96
ABNER	JNLPBA	65.27/70.80/67.92	22.88/25.17/23.97	39.91/52.51/45.36
Gimli	JNLPBA	**71.69**/**68.40**/**70.01**	32.35/23.81/27.43	42.86/43.02/42.94
NERsuite	JNLPBA_cl_	57.60/76.40/65.68	15.71/39.46/22.47	27.72/63.69/38.63
NERsuite + dict	JNLPBA_cl_	63.45/76.60/69.41	30.48/63.95/41.29	37.65/72.07/49.46
NERsuite	CellFinder_cl_	31.99/23.00/26.76	54.71/81.63/65.51	30.13/37.43/33.39
NERsuite + dict	CellFinder_cl_	60.13/33.80/43.27	**85.91**/**87.07**/**86.49**	72.40/74.30/73.34
NERsuite	Gellus	73.16/31.00/43.55	51.85/28.57/36.84	79.39/71.51/75.25
NERsuite + dict	Gellus	73.81/41.80/53.37	89.43/74.83/81.48	**91.67**/**85.47**/**88.46**

The numbers displayed in bold font represent the best performing systems for each test corpus. (Note that the evaluation on ABNER, GENIA tagger and Gimli was done with provided models solely trained on the original JNLPBA training data).

As ABNER, GENIA Tagger and Gimli are trained on the JNLPBA corpus, we focus initially on the tagging results on the JNLPBA_cl_ data set. As shown in Table 2, all tools reach similar ***F*-**scores (66–70%), ranking from highest to lowest in the order Gimli, NERsuite+dict, Genia Tagger, ABNER and NERsuite. Though the tools achieve similar ***F*-**scores, they differ more in terms of the precision/recall balance. Gimli obtains the highest precision, while NERsuite+dict achieves the best recall. All taggers considerably outperform dictionary matching on the JNLPBA_cl_ corpus.

Next we consider the cross-corpus performance of the tools. All taggers trained on JNLPBA perform worse on CellFinder_cl_ and Gellus than the dictionary method, which achieves comparatively high performance with exact matching. Remarkably high recall (>90%) is observed on the Gellus corpus with the approximate matching dictionary approach, but this inevitably comes with significantly lower precision (<15%) as a trade-off. These results support the observation of the qualitative analysis (Section 3.1) that the annotation scope of JNLPBA differs notably from that of the other two corpora in including also non-specific mentions.

Focusing on NERsuite, dictionary features improve the performance of the tool on all tested corpora. A moderate improvement in ***F*-**score is attained on JNLPBA_cl_ (<4 p.p.), and very notable increases are observed on the CellFinder_cl_ (>16 p.p.) and Gellus (>20 p.p.) corpora. The difference in the increase in performance is also likely to be related to the proportion of annotated cell lines that can be linked to the dictionary, as discussed previously (Section 3.1).

### 3.3 Cross-corpus evaluation

As noted above, the performance of the ML-based taggers drops dramatically when they are evaluated across corpora. In this section, we further explore the influence of the corpus annotation scheme on tagging performance using two additional corpora, CellFinder_cl_ and Gellus, to train NERsuite.

We first consider the intra- and cross-corpus performance of NERsuite with and without dictionary features. The results are summarized in Table 2. The performance of NERsuite and NERsuite+dict trained and tested on the CellFinder_cl_ or Gellus corpora is similar to training the tagger with the JNLPBA_cl_ corpus in that NERsuite achieves a relatively high ***F*-**score when trained and tested on datasets drawn from the same corpus. As noted in Section 3.2, the dictionary features greatly improve the performance of NERsuite on both the CellFinder_cl_ and Gellus corpora, achieving state-of-the-art results (>85% ***F*-**score).

In addition, we also analyze the performance of NERsuite and NERsuite+dict trained on either CellFinder_cl_ or Gellus corpora and tested on the other corpus. A similar result, lower ***F*-**score, is observed when the NERsuite is evaluated across corpora, regardless whether it is trained on CellFinder_cl_ or Gellus. However, the system achieves a notably higher ***F*-**score on cross-corpus evaluation if it is supported with dictionary-based features. In particular, the tagger with dictionary features performs well also on CellFinder_cl_ if it is trained on Gellus, and vice versa. The performance of NERsuite+dict is slightly higher if it is trained on the Gellus corpus. Nonetheless, the performance of NERsuite without dictionary feature trained on either CellFinder_cl_ or Gellus on JNLPBA_cl_ remains limited due to low recall.

In summary, the NERsuite tagger generalizes well if both the training and test corpora have similar specificity constraints in their annotation, such as the CellFinder_cl_ and Gellus corpora. Additionally, the results indicate that taggers trained on data not limited to a specific domain generalize better to other corpora than taggers trained on domain-specific corpora. Finally, the performance of the tagger can be notably increased by incorporating relevant dictionary features. It should be noted that the choice of using NERsuite as retrainable system is due to its relative ease in incorporating the dictionary features. Comparable performance can be expected from other CRF-based retrainable systems with dictionary-derived features.

### 3.4 Evaluation of normalization potential

Our original definition of relevant cell line mentions specifically included the need for recognized mentions to be linkable to external database identifiers so that the somatic mutations and SL interactions can be identified (Section 2.1). The CLL corpus was created specifically to assess the normalization opportunities of our approach.

As described in Section 2.2.5, we automatically introduced cell line annotations to random articles from PubMed, using the dictionary as an external reference of cell line names and following the cell line definition from CLO. The initial automatic annotation of 201 candidate cell line mentions was evaluated to have marked 147 cell line and 54 non-cell line mentions. The entity types of tagged non-cell lines include i) gene/gene product (37.03%), ii) chemical compound (14.81%) iii) organism or a part of organism name (12.96%) and other types (35.19%). Mentions of cell lines in the same sentence which were not pre-tagged were also annotated, resulting in an additional 194 cell line annotations. Altogether, there were 341 cell line name mentions in 148 sentences. From this dataset, we discarded all non cell line mentions creating CLL corpus suitable for evaluating taggers in recognizing established cell lines.

We apply NERsuite trained on different corpora both with and without dictionary features to assess the performance of the tagger on normalizable cell line names. The results, shown in Table 3, are well in line with those observed in Section 3.3 in that the best tagger is NERsuite trained on the Gellus corpus and supported with dictionary-based features. This model achieves state-of-the-art performance with good precision/recall balance for the normalizable cell lines, which is highly encouraging for the cell line recognition task in our SL application.

**Table 3.** The results of NERsuite trained on different models and applied on the CLL corpus

Trained Model	Precision (%)	Recall (%)	*F*-score (%)
CellFinder_cl_	80.00	28.15	41.65
CellFinder_cl_ + dict	86.90	69.50	77.23
JNLPBA_cl_	75.92	52.49	62.07
JNLPBA_cl_ + dict	82.93	79.47	81.16
Gellus	92.90	40.47	56.38
Gellus + dict	90.22	82.11	85.98

### 3.5 Error analysis

As shown earlier, tagging cell lines can be carried out with relatively high accuracy, however, there still remain marginal mistakes of the tagger. To shed light on the remaining challenge, we perform an error analysis by training NERsuite supported with dictionary limiting to only Gellus train data and evaluate both false positive/negative predictions on the development set. We find that most of the false negatives (10 out of 12 unique cell line mentions) are cancer cell lines which are not included in our dictionary. Thus, we can expect an increase of the overall performance if an inclusive dictionary of cell line names is used. The false positive predictions are mainly caused by the overlapping symbols from other types of entities such as diseases (e.g AGS or gastric adenocarcinoma), genes/proteins (e.g. HK2 or hexokinase 2) and animal models (e.g. LLC or Lewis lung carcinoma model). It seems to be more difficult to improve the performance of the tagger by removing the false positives as they appear in contexts similar to cell lines.

### 3.6 Large-scale application

After thorough intrinsic evaluation of all tools and training corpora/settings, we applied the best performing NERsuite model to detect cell line names in the entire publicly available literature, including 23 343 329 PubMed citations and 958 773 PMC-OA full-text articles. The processing of this large-scale set of unannotated documents will provide valuable information about the scalability of our methods and their applicability to real-world use-cases and provide a first publicly accessible literature-scale resource of normalized cell line name mentions.

We used the best model of NERsuite trained on Gellus+dict to recognize cell lines. NERsuite trained on the Gellus corpus recognized 5 181 342 mentions of cell line names in 1 003502 of a total of 24 302 102 documents. NERsuite alone took about 330 CPU core hours to tag cell line names in the entire dataset, averaging 42.5 PubMed abstracts and 1.5 PMCOA full-text documents per second. The run was parallelized on document level on a modern cluster computer.

We further normalize the recognized cell lines on this large-scale data set (Section 2.4). The results are very promising, with 91.89% tagged cell lines matching a symbol in the dictionary. Among these, 6703 tagged mentions are linked to cancer cell lines, providing a ready data set for future work on identifying SL gene pairs. As a result, we can see that the tool can also perform well on a large scale, open-domain task, such as recognizing and normalizing the cell lines in the whole literature, without compromising its performance.

## 4 Conclusions

We have presented a study of cell line mention recognition motivated by the needs of a project to identify synthetically lethal gene interactions in the literature. We analyzed publicly available machine learning-based taggers in comparison with dictionary-based tagging to identify the best-performing approach, with particular focus on mentions of specific cell line names.

We prepared resources that are essential to the development and evaluation of cell line name recognition methods. For dictionary matching, we assembled cell line-related information from Cellosaurus and other resources. For the ML-based taggers, we prepared derived versions of the publicly available JNLPBA and CellFinder corpora filtered to cell line annotation. We also created two additional manually annotated cell line corpora, Gellus and CLL. Gellus provides broad-coverage training data for ML-based taggers, and the CLL corpus, which marks sentences likely to contain normalizable cell line names, allows for extrinsic evaluation on an independently derived reference dataset.

We assessed tagger performance in both intra-corpus and cross-corpus settings, using approximate matching criteria to accommodate for differences in annotated mention spans. We additionally used the retrainable NERsuite system to evaluate the capability of dictionary features to improve tagger performance. Tools trained on JNLPBA_cl_ were found to perform well on the test set of the same corpus but to generalize poorly to other corpora. The results of cross-corpus analysis using NERsuite showed that the Gellus and CellFinder_cl_ corpora can be used to train broadly compatible taggers, with training on Gellus providing the best results overall.

The best-performing cell line name tagger, NERsuite trained on the Gellus corpus and supported by dictionary features, achieved a state-of-the-art best result of 88% ***F*-**score, far surpassing dictionary-based approaches. The tagger also achieved state-of-the-art performance when evaluated on the CLL corpus, demonstrating its suitability to recognizing cell lines that can be related to unique identifiers.

Finally, we estimated the performance of the system for SL application, tagging and normalizing cell line names on the entire publicly available literature. The system was found to scale well and evaluation of its outputs against a cell line name dictionary indicated good generalization performance to large-scale data.

To conclude, we have introduced new resources and a new system for the task of identifying specific cell line name mentions from text, achieving state-of-the-art performance. As part of an on-going project, the system applied to the entire literature recognized established cancer cell lines allowing future work in identifying synthetically lethal gene pairs from the literature. After cell line name recognition, we can form synthetic lethality relationship between cancer cell lines and human candidate genes. Depending on the data availability, we plan to continue our work on extracting the association between the cell line and candidate gene from text using rule-based and machine learning approaches. The textual support association can strengthen the synthetic lethality link between mutated gene and SL-candidate gene.
